# Reliability of Grip Strength as a Predictor of Hand Limitation Among U.S. Older Adults: How Good is Grip Strength?

**DOI:** 10.1093/geroni/igab046.3257

**Published:** 2021-12-17

**Authors:** Rachel Logue, Susan Brown, Rebecca Hasson, Matthew Davis

**Affiliations:** University of Michigan, Ann Arbor, Michigan, United States

## Abstract

Grip strength is commonly used to assess hand function in older adults and is associated with health outcomes including muscle strength, cognition, and mortality. However, the degree to which grip strength predicts an actual hand limitation is unknown. This study evaluated grip strength as a predictor of hand limitations associated with activities of daily living. Using the 2011-14 National Health and Nutrition Examination Survey (NHANES), we selected five self-reported hand-related functional limitations to classify older adults reporting one or more limitations versus those with no limitations. We identified 2,064 older adults (age≥65), 31% of whom reported a hand-related limitation. Odds ratios were used to assess the association between grip strength quartile and the likelihood of a hand limitation while controlling for sex, race/ethnicity, education level, income, and pain. Receiver operator curves were used to evaluate the degree to which grip strength discriminates between those with limitations versus those without. Older adults with very low grip strength (lowest quartile) were more likely to have at least one limitation (OR:6.1, 95% CI:3.2,11.8) than those with high grip strength (highest quartile). However, receiver operator curves suggested grip strength only modestly discriminated hand limitations (area under curve:0.71). While self-reported hand limitations were associated with lower grip strength, it was a relatively poor predictor of hand impairments among older adults. This study suggests grip strength may not predict hand function as well as previously thought. Better assessments are needed to adequately evaluate upper extremity impairments to help older adults maintain functional independence.

